# Volcano alert level systems: managing the challenges of effective volcanic crisis communication

**DOI:** 10.1007/s00445-018-1219-z

**Published:** 2018-04-13

**Authors:** C. J. Fearnley, S. Beaven

**Affiliations:** 1 Department of Science and Technology Studies, University College London, Gower Street, London, WC1E 6BT UK; 2 Disaster Risk and Resilience Group, Department of Geological Sciences, University of Canterbury, Private Bag, Christchurch, 48000 New Zealand

**Keywords:** Volcano alert level systems, Communication, Volcano observatories, Volcano monitoring, Decision-makers

## Abstract

Over the last four decades, volcano observatories have adopted a number of different communication strategies for the dissemination of information on changes in volcanic behaviour and potential hazards to a wide range of user groups. These commonly include a standardised volcano alert level system (VALS), used in conjunction with other uni-valent communication techniques (such as information statements, reports and maps) and multi-directional techniques (such as meetings and telephone calls). This research, based on interviews and observation conducted 2007–2009 at the five US Geological Survey (USGS) volcano observatories, and including some of the key users of the VALS, argues for the importance of understanding how communicating volcanic hazard information takes place as an everyday social practice, focusing on the challenges of working across the boundaries between the scientific and decision-making communities. It is now widely accepted that the effective use, value and deployment of information across science-policy interfaces of this kind depend on three criteria: the scientific credibility of the information, its relevance to the needs of stakeholders and the legitimacy of both the information and the processes that produced it. Translation and two-way communication are required to ensure that all involved understand what information is credible and relevant. Findings indicate that whilst VALS play a role in raising awareness of an unfolding situation, supplementary communication techniques are crucial in facilitating situational understanding of that situation, and the uncertainties inherent to its scientific assessment, as well as in facilitating specific responses. In consequence, ‘best practice’ recommendations eschew further standardisation, and focus on the in situ cultivation of dialogue between scientists and stakeholders as a means of ensuring that information, and the processes through which it is produced are perceived to be legitimate by all involved.

## Introduction: communicating during volcanic crises

Over 80 volcano observatories across the globe are tasked with monitoring and communicating timely and useful information about the behaviour of a volcano (WOVO 2017). This assessment and communication role is structured around volcano early warning systems, constituting a range of communication techniques developed by volcanologists and policy makers to provide information to populations at risk from volcanic hazards and to allow them to seek safety, both locally and regionally (Peterson et al. 1993). Such information is crucial to the work of decision-makers responsible for safety (Mileti 1999), who need insight into when and where the volcano may erupt; the magnitude, style and duration of the eruption; likely hazards and expected location; and the effects of volcanic hazards at the local, regional and global scale (Mileti and Sorenson 1990). One of the key elements of a volcano early warning system, and the most widely disseminated through the media, is a volcano alert level system (VALS), which is summarily defined as a “series of levels that correspond generally to increasing levels of volcanic activity” (Gardner and Guffanti 2006, p. 2). Globally, scientists are typically responsible for determining and disseminating the appropriate alert level, although some countries have differing responsibilities within the actors involved (WOVO 2018). Deploying VALS requires that the scientists involved consider how best to impart the scientific uncertainty associated with monitoring data to user groups, in sometimes emotive conditions (Harris 2015; Leonard et al. 2008), whilst also contending with the technological constraints of the monitoring techniques available, budget limitations and political sensitivities (Potter et al. 2017; Fearnley et al. 2018). VALS research to date has focused almost entirely on improving the credibility and consistency of information that comes from scientists operating in a national context. Largely as a result, VALS have been subject to increased standardisation at national and international levels with the explicit aim of improving information (Potter 2014; Fearnley et al. 2012).

It has been well established that VALS are designed to provide a ‘bridge’ between the scientific data on risk gathered through the monitoring process and the mitigation decisions and actions involved in the practical management of and response to the relevant hazards (Fearnley 2011; Gardner and Guffanti 2006). There has been very little research to date, however, concerned with the use of VALS to facilitate the communication of the scientific assessment of risk to those required to make practical management and response decisions. Recent exceptions have focused on the issues that arise around the distribution of tasks and decision-making responsibilities between the volcano scientists responsible for deciding alert levels, and the decision-makers who rely on the alert levels when making decisions during volcanic crises that have significant social consequences (e.g. Andreastuti et al. 2017; Hill et al. 2017; Newhall and Solidum 2017; Potter et al. 2017). Fearnley (2013) established that in practice, the high levels of both scientific uncertainty and risk so characteristic of volcanic activity have required that scientists consult locally and take social and political factors into account when deciding alert levels. Fearnley concludes that more transparently deliberative approaches that bring scientists and decision-makers together to agree on alert levels would have the potential to legitimise a greater level of coproduction of knowledge, and increase shared understanding of the uncertainties and risks involved on all sides. More recently, Papale (2017) appears to take issue with this suggestion. Citing the “principle of separation of roles”, he proposes instead that “scientists should base their evaluations exclusively on scientific knowledge, providing decision-makers with clear, unambiguous information that they can use to fulfil their societal and political mandates” (Papale 2017, p. 4). He maintains that this information should consist of probabilistic forecasts, which he finds to be “in a form most suited to provide decision-makers with the realistic picture for their subsequent decisions” (Papale 2017, p. 4). Papale concludes that VALS should be replaced by a “rational approach, in which varied expertise is harnessed in a coordinated effort, uncertainties are fully recognised and quantified, communications are unambiguous, and responsibilities reflect the social role and societal mandate of all groups involved” (Papale 2017, p. 4).

The coordinated effort that Papale invokes here has been the goal of a wide range of science/policy collaborations over the last three decades. But a large body of research in sociological and science and technology studies has found that systemic factors make it difficult to achieve coordinated efforts in which uncertainties are recognised and quantified, communications are unambiguous, and responsibilities are clearly socially mandated (Beck 1992; Funtowicz and Ravetz 1994; Nowotny 2015; Nowotny 2005; Pielke 2007; Stirling 2007). The problems stem from the way that science communication actually works on an everyday basis (Douglas and Wildavsky 1983; Jasanoff 2004; Wynne 1996; Shackley and Wynne 1996). Scientific credibility criteria are an effect of how meaning has been created, disseminated and recreated (Gieryn 1999). As early as 1980, Hall (1980) established that it is not enough to focus narrowly on decision-making per se; it is equally important to understand frameworks of knowledge, relations of productions and technical infrastructure involved in both the provision and reception of information. Two decades later, Cash et al. pointed out a large body of work that had:

identified the importance to effective science advising of ‘boundary work’ carried out at the interface between communities of experts and communities of decision makers. This work highlights the prevalence of different norms and expectations in the two communities regarding such crucial concepts as what constitutes reliable evidence, convincing argument, procedural fairness, and appropriate characterisation of uncertainty. It points out the difficulty in effective communication between the communities that results from these differences, and stresses the importance for effective advising of explicit development of boundary-spanning institutions or procedures (Cash et al. 2003, p. 8086).

Cash et al. (2003) drew from more than 30 case studies to confirm that the use of institutions or procedures that span this interface between scientific and decision-making communities have been necessary to establish the usability and potential influence of scientific knowledge. The effective use, value and deployment of information across this interface depend on three interlinked criteria: the scientific credibility of the information, its relevance to the needs of stakeholders and the legitimacy of both information and the processes that produced it. Translation of scientific concepts and terminology into accessible everyday language is required to ensure that everyone involved understands why and how information is scientifically credible (Cash et al. 2003). Multi-valent communication among all involved is required to ensure that all involved, including scientific communities, fully understand relevance to stakeholder needs. The legitimacy of the information relies on the perception that the interests and influences of all those involved, including both scientific and end user groups, are included and balanced; legitimacy relies on transparency, and is enhanced by mediation arrangements.

The research that Cash et al. (2003) built on had already established that coordinated efforts of the kind invoked by Papale (2017) necessarily occur at the hybrid, dynamic interface between scientific and other communities, where the strategic demarcation of scientific and other tasks involves inevitable crossover (Guston 2001; Jasanoff 2011a, b; Parker and Crona 2012; Drimie and Quinlan 2011). In this article, we start from this understanding; it is not possible to restrict scientists to science when they are communicating scientific information across the boundary that divides science and non-science knowledge domains, and neither is it best practice.

Cash et al.’s (2003) framework has been influential in a range of topic areas concerned with large, complex issues. First gaining traction in environmental management (Cash et al. 2003; Sternlieb et al. 2013; Pesch et al. 2012; Parker and Crona 2012; Crona and Hubacek 2010; Van den Hove 2007), this framework is now widely used in biodiversity debates (Koetz et al. 2008, 2012; Perrings et al. 2011; Sarkki et al. 2013), and in sustainable development (Hotes and Opgenoorth 2014; Runhaar and van Nieuwaal 2010), climate change (Lee et al. 2014; Hoppe et al. 2013; Friman and Strandberg 2014; Iyalomhe et al. 2013) and public health fields (Drimie and Quinlan 2011; Casale et al. 2009; Creech and Willard 2001). There have been few explicit applications of Cash et al. (2003) in the disaster risk reduction context. Recent exceptions have focused on the use of boundary spanning institutions and procedures for disaster risk management across scales (Djalante 2012), to coordinate national research funding (Beaven et al. 2017) and research support for disaster response and recovery agencies (Beaven et al. 2016), and to implement tsunami early warning systems in the Indian Ocean region (Thomalla and Larsen 2010).

In this article, we draw from this larger body of work, using Cash et al.’s (2003) framework to analyse the way that VALS has worked as a communication device. The aim is to build on the work of Fearnley (2013) with a further clarification of the value and limitations offered by VALS as a boundary spanning initiative, to demonstrate the utility of this framework in the volcano alert context and to offer recommendations concerning ‘best practice’ deployment.

## An evolution of volcano alert level systems globally

VALS (also referred to as status levels, condition levels or colour codes) are used globally to provide volcanic warnings and emergency information in relation to volcanic unrest and eruptive activity based on data analysis or forecasts. VALS can be thought of as a ‘black box’. In this concept developed by Bruno Latour (1987), although both inputs and outputs are known, the inner workings remain hidden, in that they become so widely accepted by both the scientific community and society that they are no longer open for debate. According to Latour, this is “the way scientific and technical work is made invisible by its own success. When a machine runs efficiently, when a matter of fact is settled, one need focus only on its inputs and outputs and not on its internal complexity. Thus, paradoxically, the more science and technology succeed, the more opaque and obscure they become” (Latour 1999, p. 304). VALS appear to be simple (one reason they are so easy to black box) and so tend to be treated as such. As Thomalla and Larsen (2010) note, however, the implementation of warning systems “consists of a wide range of social and organisational processes” and rely on “chaotic patchworks of communication that require multiple iterative coordinative actions between agencies, officials and citizens” (p. 252). To understand the challenges faced by those implementing VALS, it is necessary to open the black box and examine the social and organisational processes that it obscures. This, in turn, requires an understanding of the way that VALS have come about.

VALS have evolved over the last 40 years in response to a series of crises that have triggered scientists and civil protection to devise systems to convey states of volcanic unrest. To date, no complete history of the evolution of VALS globally has been compiled. In addition, there is no catalogue of VALS globally, nor any authoritative source of alert levels that is official and endorsed. The current VALS Working Group^1^ that sits within the World Organisation of Volcano Observatories (WOVO) Commission is working towards these goals, and to provide a more informed understanding of VALS to help volcano observatories make more informed decisions about the design, implementation and integration of their VALS. Fearnley et al. (2017) provide a broader overview of the evolution of thinking around volcanic crisis communication over the last century, bringing together studies on relevant case studies, which include a focus on early warning systems that provide a vital context to the development of VALS.

The development of VALS began in the 1980s, in response to the Mt. St. Helens eruption (USA) in particular. Between June 1980 and October 1986, this volcano continued to erupt in the form of a dome-building phase punctuated frequently by dome explosions (Swanson and Holcomb 1990). This cyclic activity allowed the newly formed Cascades Volcano Observatory (CVO) to develop accurate warnings as far as 3 weeks in advance for 19 of 21 explosions (Bailey and USGS 1983). Increasing confidence for many scientists in their ability to provide precise predictions, this high rate of accuracy provided the impetus to develop a VALS for use at CVO. In 1985, the United Nations Disaster Relief Organisation (UNDRO) published a report on ‘Volcanic Emergency Management’. It features one of the first examples of a VALS, called “stages of alert of volcanic eruption” (UNDRO 1985, p. 54). Each progressive alert level reflects increasing indicators that the volcano is about to erupt and provides an approximate period and a recommended disaster manager response. From this point on, VALS have all followed this linear progression whereby alerts rise with perceived increasing levels of danger. The UNDRO report also offers strong guidance in relation to using public announcements that have been decided prior to any emergency to limit panic in volcanic crises, emphasising the need for the public to be made aware of the arrangements for receiving information. These details vary in each place, region and country, according to the different “political and social structure of the community and the technical means available. It is therefore difficult to lay down any detailed guidelines for public information and warning” (UNDRO 1985, p. 55). The report also highlighted the importance of local context and the need to develop an idealised VALS for countries to adopt or adapt if they required. Possibly, because of the importance of local contingencies, literature on VALS since 1985 has remained limited until the 2000s, with some grey literature written by various volcano observatories, institutions and individuals.

By the late 1980s, VALS were being used by observatories in a number of countries, when a new type of VALS was devised to communicate to the airline industry. During the eruption of Redoubt on December 15, 1989, a Boeing 747 aircraft lost power in all four engines. Forced to glide, it had lost 4 km in altitude when it restarted the engines only 1 km above nearby mountain peaks (Brantley 1990). In response to this event, the Alaska Volcano Observatory (AVO) was developed, and began using its aviation colour code system in February 1990 during the ongoing Mt. Redoubt eruption. Unlike the VALS that preceded it, this code needed to specifically communicate ash hazards. The AVO Scientist in Charge (SIC) and head of the US Geological Survey (USGS) Volcano Hazard Program (VHP) came up with the idea, as recorded during email correspondence from the AVO SIC in 1990 (FOIA^2^ archives) to senior VHP scientists at Menlo Park:

[We] desperately needed a simple device to communicate to the airline industry the activity, or anticipated activity of Redoubt volcano. I sprung it on the large AVO group (maybe as many as 30 people mostly from CVO, Menlo Park, etc.) at the next morning’s staff meeting in Anchorage. Given the fact that we had geologists, seismologists, lahar specialists, tephra people etc., the proposed warning scheme almost immediately ballooned to a 6 x 6 matrix (!) so as to satisfy everyone. After the meeting was over, we went back to the original 4-colour scheme.

The success of the AVO Aviation Colour Code following the 1996 Pavlof eruption (Roach et al. 2001) and 1989 Mt. Redoubt eruption (Casadevall 1994) led to its adoption in 2005 by the International Civil Aviation Organisation (ICAO). It is now an internationally recognised, used and increasingly enforced VALS.

During the 1990s, several significant eruptions occurred enabling the opportunity to design and implement specifically designed VALS including at Mt. Pinatubo, Philippines in 1991 (Newhall and Solidum 2017; Newhall and Punongbayan 1996); Rabaul, Papua New Guinea in 1994 (McKee et al. 2017); and Soufriere Hills, Montserrat from 1995 to present (Aspinall et al. 2002). This resulted in numerous VALS designed around specific volcanoes. These crises, among others, led to a focused discussion around the management of volcanic crises, the role of various warning systems and of the scientists involved, resulting in the publication ‘Professional conduct of scientists during volcanic crises’ emanating from the 1999 IAVCEI Subcommittee for Crisis Protocols (Newhall 1999).

Since the turn of the century, increasing standardisation across national VALS has occurred, facilitating national adaptations to better fit volcanism type and national emergency management protocols. The growing number of nationally adopted VALS is illustrated, for example, by the 2006 standardisation of USGS VALS, in which three different VALS were replaced by the standard VALS now used at all five volcano observatories (Fearnley 2011). Similarly, until recently, New Zealand operated two systems: one designed for the hazards expected at frequently active cone volcanoes and another for reawakening volcanoes. Both were based on numbered levels (from 0 to 5) (GNS 2010). In 2014, however, these were revised into a single VALS for ground-based hazards (Potter et al. 2017). Many observatories continue to deal with more than one VALS during a crisis. Both the US and New Zealand alert levels are decided by the current activity of a volcano; they do not provide action or advice to users for mitigative action. In contrast, the Japanese VALS states the measures to be taken by specifying areas of danger, indicating the extent of evacuation and outlining expected volcanic activity (Japan Meteorological Agency 2010). In Indonesia, the Center for Volcanology and Geological Hazard Mitigation (CVGHM) uses VALS to outline the potential impact of the volcanic behaviour on surrounding communities, integrate capacity building in communities and assist in the implementation of actions during volcanic eruptions according to alert level (Andreastuti et al. 2017). Montserrat Volcano Observatory has designed an VALS whereby certain designated zones on the island are assigned an alert level that determines access restrictions to those zones. These examples demonstrate the diversity in the style, design and use of VALS to cater for the particularly requirements of each observatory; in the case of Monserrat, the need to make sure people move to safe zones or avoid dangerous ones (Donovan and Oppenheimer 2015; Donovan et al. 2012). VALS used in developing countries are more likely to provide advice on mitigative action or evacuations to civil authorities and emergency managers. The many factors involved in designing a VALS include what information is provided, whether actions are recommended, the style of warning (actual or forecast) and the number of VALS used. Different countries may also offer differing capacities for decision-making in response to volcanic activity, moving from an extreme end-member where the alert level de facto establishes actions, through to the public authorities making the decision in isolation from the scientists.

WOVO states that despite often worldwide interest in the status of any given volcano, “with the exception of colour codes for aviation, currently there is no standardised international volcano alert levels system” (2018). This is due to the “wide variation in the behaviour of individual volcanoes and in monitoring capabilities, and different needs of populations, including different languages and symbolism of colours or alert levels” (2018). The WOVO recognises the importance of local contingency, but also the fact that the aviation sector requires a standardised tool they can understand regardless of which airspace they are flying through.

Despite these variances, the possibility of developing a globally standardised VALS for ground hazards has been considered. After exploring this possibility at volcano conferences in the late 1990s and early 2000s, Scott (2007) concluded that there cannot be international uniformity in VALS due to the wide range in volcanic eruptions and hazards, and the recurrence of activity that requires a wide variety of needs to be catered for. Scott questioned the standardisation process, asking if it actually “undermines the important function they achieve” (Scott 2007, p. 90).

Although VALS provides essential volcanic information to decision-makers, only a handful of journal publications specifically review the implementation of VALS, discussing the way they are operated and analysing strengths and weaknesses. In the first of these key studies, Metzger et al. (1999) reviews the impact a ‘yellow alert’ issued in Quito, Ecuador, for the Guagua Pichincha volcano during unrest in 1998. They demonstrate that VALS become complicated precisely because they include stages of alert whereby each alert has an impact on vulnerable societies; because they are constantly changing, VALS are difficult systems to understand. The second study, by De la Cruz-Reyna and Tilling (2008) focuses on the introduction of a ‘Volcano Traffic Light’ alert system for Popocatépetl volcano in Mexico, which the USGS assisted in designing and implementing. This VALS has seven levels of alerts for emergency-management authorities, but only three levels for the public (green-yellow-red). This paper highlights the fact that there is a further disparity within national VALS, as some countries, such as Mexico, have different VALS for the decision-makers and for the public. Both papers highlight the need for VALS to be locally adapted and demonstrate the difficulties of using a linear VALS when volcanic crises can occur for long periods, causing warning information requirements to change as seen in the Metzger et al. study of Guagua Pichincha Metzger et al. (1999). Following these two initial publications, Fearnley published on the use, standardisation and role of VALS at the USGS (Fearnley 2011; Fearnley et al. 2012; Fearnley 2013), and there have been a number of publications on the use of VALS in New Zealand (Potter et al. 2017; Leonard and Potter 2015; Potter 2014; Potter et al. 2014). Winson et al. (2014) published an analysis of the issuance of volcanic alert levels during volcanic crises stating that between 1990 and 2013, 30% of volcano warnings issued worldwide prior to the eruptions of VEI3 or larger accurately reflect the potential ‘hazard before eruption’. Their findings suggest that the issuance of information prior to an eruption is a complex, multifaceted problem, and the evaluation of what constitutes ‘success’ is itself a complicated question (they ask is 30% successful)? Aspinall et al. (2006) reviewed the role of using hidden multi-state Markov models with multi-parameter volcanic data to provide empirical evidence for alert level decision-support, thus using Baysian statistics to assign an alert level. Some observatories also tie in monitoring thresholds and criteria to determine their alert levels. Papers that focus on more recent case studies include Kato and Yamasato (2013) that discuss the 2011 eruptive activity of Shinmoedake volcano and the challenges of not having clear precursory activity to provide an alert. García et al. (2014) discus the volcanic alert system (VAS) developed during the 2011–2014 El Hierro (Canary Islands) volcanic process, focusing on the monitoring network, the software tools for analysis of the monitoring parameters, the VAL management and the assessment of hazard, providing a useful system that could be useful to others.

Finally, a study by Donovan et al. (2018) investigating risk perception at Popocatépetl volcano in Mexico (this volume) highlights important links between warnings and trust and the perceived motivation of particular groups, perceived trust and perceived knowledge. Following an online survey carried out from 2012 to 2014, their findings indicate that volcanic warnings and, more specifically, VALS are in fact effective, with greater level of risk perception in the public during raised alert levels as a result of higher activity, aiding to the communication and understandings of risk. Donovan et al. (2018) highlight the “importance of considering the social impact of warnings” (p?) whilst also emphasising that VALS act as a boundary object allowing “the translation of risk information into terms that people can understand” (p.?) between science and society, serving different functions on each side of that boundary. The key thing to note is that whilst the perceived knowledge of the volcano was the most important factor in explaining trust, VALS have considerable and complicated social impact. Studies such as these illustrate that there is still significant work to be done in exploring and evaluating VALS.

In summary, VALS differ greatly between countries, with some including only descriptions of the level of physical phenomena (e.g. differing criteria of volcanic unrest and size of eruption), whilst others include hazards, potential impacts and risk mitigation actions (including evacuations). Some include forecasting, whilst others do not. Designing new VALS and evaluating or revising existing systems requires an understanding of these options. These processes benefit from being able to draw upon the experiences of others in similar situations, and the related theory of risk (and crisis) communication. With increasing levels of technology and communications methods (such as social networking), it is imperative that VALS used by volcano observatories around the world retain their credibility and trust, and work to serve legal, political and local community requirements. This requires further investigation in understanding how uncertainties are conveyed and represented within the VALS and how these are perceived by key decision makers, as discussed by Fearnley (2013) and Fearnley et al. (2017). It is also important to note that the original intent of VALS may vary in different countries and that intent is very different from the reality of the task, resulting in VALS evolving in different ways over the years, in part to deal with changing technologies, growing and more complex societies and differing legal and institutional remits and protocols.

Another key distinction that has developed as VALS have been designed and implemented around the world concerns two distinct institutional roles awarded to volcano observatories. In many countries, volcano observatories are classified as state or gederal services. This is the case for example in New Zealand and the USA, where such observatories are part of national research agencies required to provide a public interface (GNS and USGS respectively), in Japan and Iceland, where observatories are run from within National Meteorological Agencies, and in Mexico, where volcano observatories are run from within the Centro Nacional de Prevención de Desastres. These institutional arrangements and associated legal remits predispose the relevant observatories to interface with end users, since they are required to provide and prioritise public functions (typically of civil protection). However, in other countries, volcano observatories are situated within institutes with a predominantly scientific focus. In Ecuador, Italy and France, for example, the Instituto Geofisico, the INGV and Le Institut de Physique du Globe de Paris (IPGP) (respectively) are positioned in the science domain, which requires a primary focus on producing quality science. It is possible that institutional remits that require this more exclusive focus on scientific goals may result in VALS design and implementation less attuned to an interface function between scientific and end user communities. Papale’s (2017) defence of a clear division between scientific and operational functions, for example, could be interpreted in the light of this distinction, since he is based in an institute that focuses on producing scientific, rather than public service outcomes. We raise this point to note that this article draws from research conducted exclusively with USGS scientists and the associated range of USGS VALS end-users. Since the USGS is required to provide a public interface function, these research participants are likely to see the role of the volcano observatory framed in terms of direct utility serving society.

This distinction aside, all volcano observatories need to use a range of communication strategies in addition to VALS to share information about changes in volcanic behaviour and potential hazards with stakeholders. These strategies are explored in ‘Observing the Volcano World: Volcanic Crisis Communication’ (Fearnley 2018), which brings together knowledge and first-hand experience from a wide range of stakeholders to provide a platform for understanding how volcano crises are managed in practice. Areas explored include (i) the need to understand the multiple hazards involved in a volcanic crisis, (ii) lessons learned from past crises and (iii) the tools available for effective communication during a crisis. The chapters in this publication address the idealised program to reduce volcanic risk devised by Tilling (1989), by exploring the divide between volcano scientists who are responsible for providing the best possible scientific information and advice, and those who have knowledge of other key factors (e.g. socio-economic, cultural, political and mass media) and the authority to make final decisions regarding mitigation measures. This divide has been exacerbated by the 2011–2015 L’Aquila court hearings, in which five scientists and two emergency managers were tried for allegedly making poor judgements on uncertainty that affected both their communications to the public and the risk management actions the public took in response (Bretton et al. 2015; Alexander 2014; Aspinall 2011). All VALS have been designed—and are required—to bridge this challenging divide.

## Using VALS to communicate across the science/policy boundary

VALS are complex to define, in part because differing nations use their alert levels for differing purposes. In New Zealand, VALS are focused on the phenomenon; in the USA, they are focused on the hazard, whilst in Indonesia and Japan, VALS are focused on the risk and response required. Irrespective of focus, however, the defining feature of VALS as a communication device is that each has to work across different disciplines, user and stakeholder groups, and a range of institutions, each with their own protocols and policies. This means that VALS functions as a ‘boundary object,’ inhabiting ‘intersecting social worlds’ and satisfying the ‘informational requirements’ of each of these worlds (Star and Griesemer 1989, p. 393). As originally defined, this sociological concept applies to scientific objects that

are both plastic enough to adapt to local needs and the constraints of the several parties employing them, yet robust enough to maintain a common identity across sites. They have different meanings in different social worlds but their structure is common enough to more than one world to make them recognisable, a means of translation (Star and Griesemer 1989, p. 393).

In addition to serving as a means of translation, and in this way enhancing understanding of why information is (or is not) scientifically credible, boundary objects (Cash et al. 2003)

- Are more likely to produce relevant, usable information because they engage end-users early.
- Can increase scientific credibility by involving different types of expertise.
- Can enhance legitimacy by providing transparent access to the processes of information production to multiple stakeholders.

Applying this concept to analyse the use of VALS offers to clarify the role of underlying drivers in the way that volcanic crisis communication has operated in practice both prior to and during volcanic emergencies from 2007 to 2009. The USGS VHP is used as a case study. Operating across five observatories, which have been established to monitor and research volcanic phenomena and risk that manifest a wide range of behaviours in different parts of the world, this program engages with a range of different cultures, communities and user and stakeholder groups. Between 2007 and 2009, 93 semi-structured interviews^3^ were conducted with both observatory scientific personnel and relevant user groups associated with the AVO, CVO, Hawaii Volcano Observatory (HVO), Long Valley Volcano Observatory (LVO) (re-established in 2012 as the California Volcano Observatory CalVo), and Yellowstone Volcano Observatory (YVO). In addition to scientists employed in observatories, interview participants were drawn from user groups including other federal agencies such as US Emergency Managers (country and state levels), the National Weather Service, US Forest and National Park managers, the Federal Aviation Administration, Volcano Ash Advisory Centre staff, local town managers and police and also included local and national media (for a full list, see Fearnley (2011, pp. 108–109)).

A qualitative multi-sited ethnographic study was adopted to trace the development and use of communication tools across and within multiple sites of activity (the volcano observatories and user groups) by following connections, associations and putative relations. The study examined “multiple sites of observation and participation that cross-cut dichotomies such as the ‘local’ and the ‘global’” (Marcus 1995, p. 95) to explore broader trends and uses of volcanic communication tools. This methodology provided a framework to establish and evaluate warning communications in volcanic observatories by providing a “way to engage with scientific and technical practice in complex allegiances that go beyond description and critique” (Hine 2007, p. 668). Interviews were used as an ethnographic tool to gain insights into how each observatory operated in the context of communicating volcanic hazards, supported by ethnographic observations from site visits and meetings, and documental analysis of the communication products at the case study. The interviews focused on the personal perspectives of scientists and users involved in the design and implementation of the VALS as these related to what interviewees considered the definition and purpose of VALS, how meaning was created within the VALS, the role and interactions of scientists and user groups using various communication techniques and their assessment of the effectiveness of various communication techniques.

It is useful to reiterate that this research, and the methodology used, did not conceive of information as a set of fixed meanings that are imparted and interpreted with various degrees of accuracy via the medium of tools that can ‘distort’ this accuracy. Communication was viewed as a social practice, where objects and events are invested with meanings that are selectively deployed, and that can be articulated in various ways depending on the medium. Whether textual, image-based or embodied, the medium plays a profound role in the way that meaning is constructed, insofar as the way in which information is represented can facilitate its manifestation as an uncomplicated ‘fact’, as in for example a basic warning system, or as a complex, multi-dimensional narrative that persuades an audience as to its veracity, as in a newspaper report or film. The manner in which a medium ‘works’ to convey meaning has in turn profound implications for the way in which information is garnered and made sense of by a wide range of individuals who on the one hand have a unique perspective predicated on their personal experience to date, but on the other have also been ‘schooled’ to read various mediums in particular ways. From this point of view, a narrow focus on the accuracy (or not) of information misses the point of how communication actually works on an everyday basis, insofar as such criteria are themselves an effect of how meaning has been created, disseminated and recreated. Constructions and interpretations can be more robust than others to the extent that they more usefully convey the variety of factors at work in a situation or event, such as the unfolding of a volcanic hazard, and can form the basis for a more effective response to this. It is on these terms, however—specifically, the use value of information and its successful deployment—that an evaluation of a warning system must be based.

## Standardised VALS and supplementary information in the USGS

The USGS supports five volcano observatories, as outlined above and has evolved a number of nationally standardised communication systems such as the VALS, and tools that communicate specific information to different user groups. Star and Griesemer (1989) link the development and maintenance of boundary objects to method standardisation, noting that these are the two key processes involved in effectively “developing and maintaining scientific coherence across intersecting social worlds” (Star and Griesemer 1989, p. 393). A number of features make standardisation attractive. First, it improves the ‘doability’ of work. Fujimura argues that doability enables scientists to “constrain work practices and define, describe, and contain representations of nature and reality” and enables a “dynamic interface to translate interest between social worlds” (Fujimura 1987, p. 205). Second, it enables simpler procedures for people to learn from and carry out. Third, in a number of spheres, particularly medical and ethical, it provides answers to public concerns relating to processes or procedures (Hogel 1995). Fourth, standardisation provides political ordering and control. In summary, standardisation offers a tool to communicate in compatible ways (via language or protocols), ensure minimum quality and provide a reference point (David and Greenstein 1990).

**Fig. 1:**
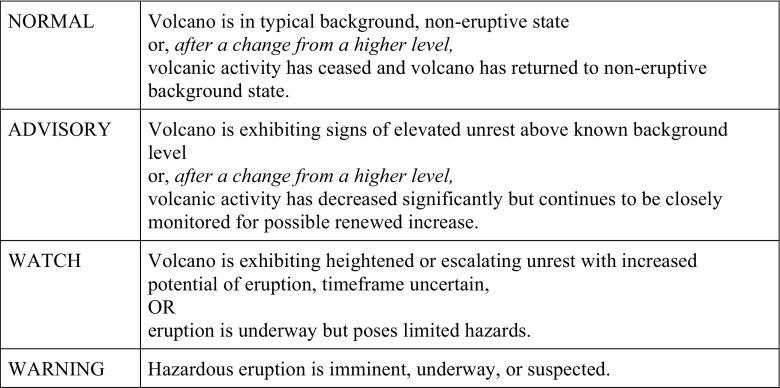
Volcano alert levels (Gardner and Guffanti 2006, p. 2)

**Fig. 2:**
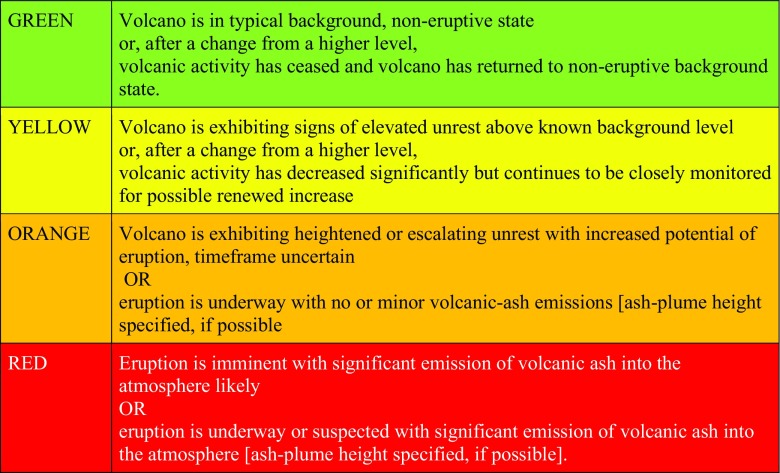
Aviation Colour Codes (Gardner and Guffanti 2006, p. 3)

In 2006, the USGS adopted two standardised VALS (one for ground-based hazards and the other for aviation ash hazards) replacing pre-existing VALS that were locally developed at each volcano observatory (Gardner and Guffanti 2006) (see Figs. 1 and 2). The Aviation Colour Code developed by AVO was adopted as the international warning system for volcanic ash by the ICAO in 2006, and subsequently is the first globally standardised VALS in the world.

The USGS established four key ‘standard’ requirements for VALS, which were to “(1) accommodate various sizes, styles, and duration of volcanic activity; (2) work equally well during escalating and de-escalating activity; (3) be equally useful to both those on the ground and those aviation; and (4) retain and improve effective existing alert notification protocol” (Gardner and Guffanti 2006, p. 1). Notably, three of these requirements are directly concerned not with scientific information as such but rather with function—the effectiveness and usability of the VALS as a communication tool. Ongoing standardisation processes were driven by a combination of factors: internationally by the adoption of the internationally used ICAO aviation colour code; nationally by the social context of the post-9/11 USA, which shaped the broader emergency management policy; at state level by the requirement to have consistent VALS and alert level terminology to prevent confusion; and internally within the USGS, to provide a more consistent and clear message. These standardisation processes are discussed in more depth in Fearnley et al. (2012).

**Fig. 3:**
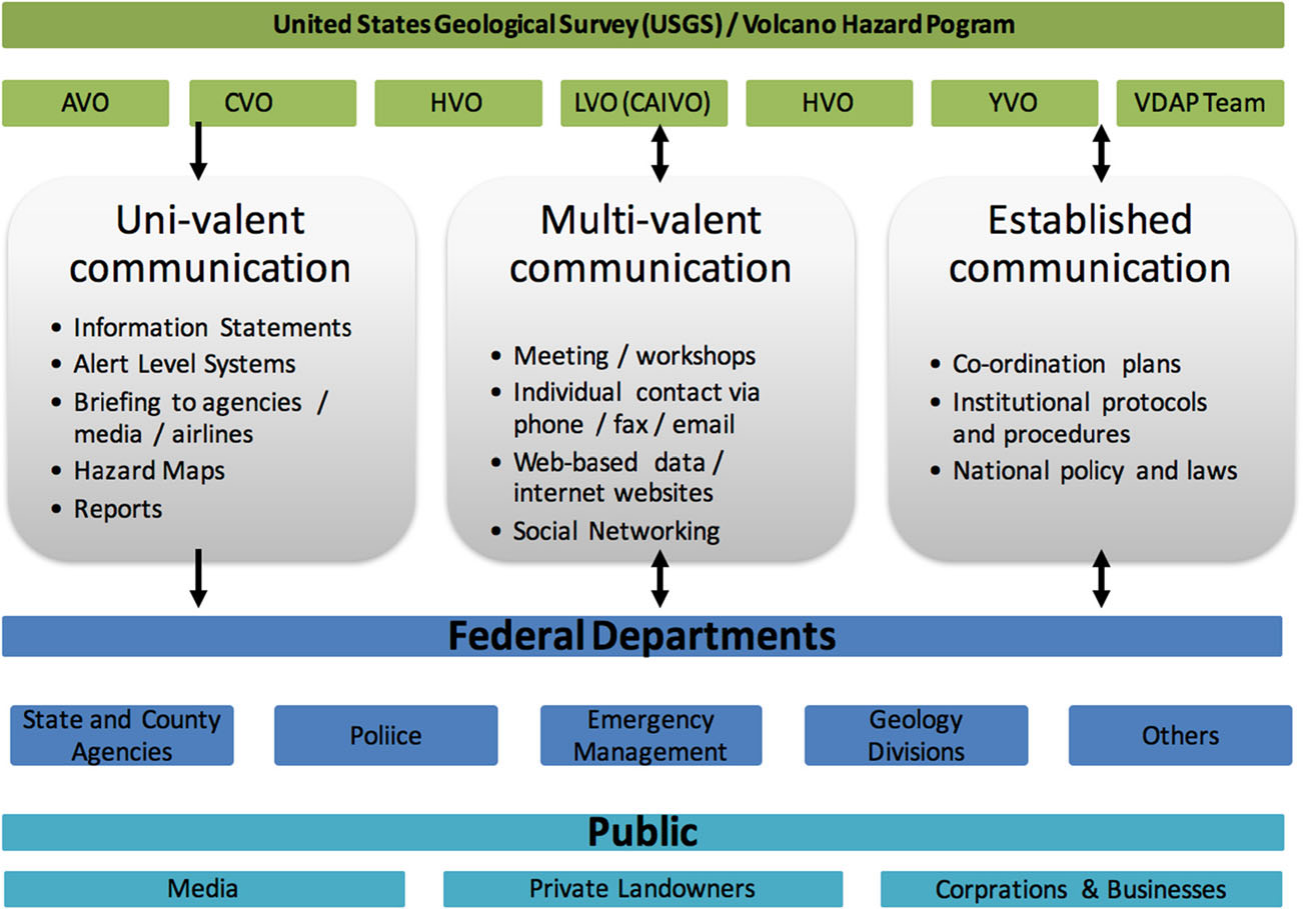
Classifying communication tools employed between the USGS and other users during volcanic crises (adapted from Fearnley (2013, p. 1896))

VALS, however, are not disseminated in a vacuum. As Gardner and Guffanti (2006) put it, “by themselves alert-level terms and code colours do not convey enough information for those in affected communities and aviation to make decisions regarding specific courses of action” (p. 4). Prior to standardisation in 2006, the range of VALS created by individual observatories were supported by a range of communication techniques, including telephone call-down lists, meetings between the relevant actors, coordination plans, media talking points and personal communication between the decision-makers. These can be described as either information provision or knowledge sharing, depending on whether they allow for two-way communication (Fig. 3).

**Fig. 4:**
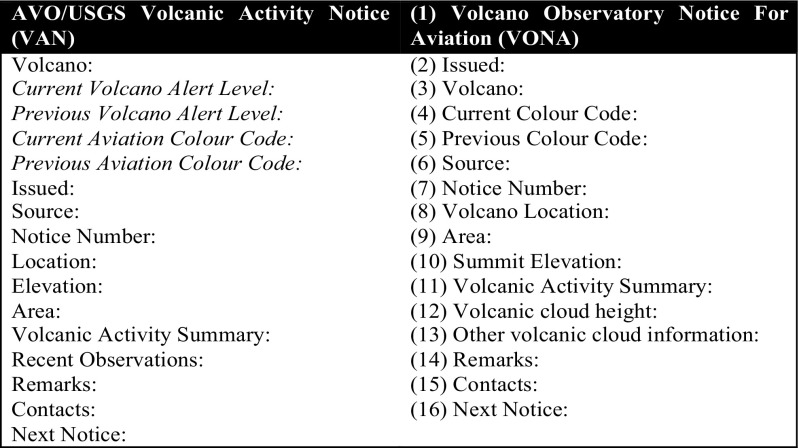
The framework for the newly devised VANs and VONA messages used in the USGS, following the standardisation of the VALS

After 2006, a number of knock-on changes were made to retain and improve effective existing alert notification protocols. Led by AVO (which is well resourced to review and launch computer and web-based products), the VHP attempted to develop a more geographically consistent communication strategy by consolidating previous uni-valent techniques into three categories (USGS 2017):

1. Event-driven (urgent) messages designed specifically to fulfil users’ requirements, using a Volcano Observatory Notice for Aviation (VONA) and a volcano alert notification (VAN) for ground hazard focused users (see Fig. 4).
2. Time-driven (scheduled) status messages.
3. General information statements.

Additional information is provided to comply with the US government Common Alerting Protocol (CAP) guidelines required by all emergency federal agencies and activities to facilitate quick warnings (Oasis 2008). These messages are available via a Volcano Notification Service (VNS), modelled on the USGS Earthquake Notification System (Fig. 4). Standardisation in this case is predicated on a more systematic database with specific input fields. All the data is available on the VHP website with alert levels assigned on a single map using a specially designed code to represent both the volcano alert level and colour code (see http://volcanoes.usgs.gov). Flexibility is derived from the fact that different end products are geared to different users (aviation versus ground) who have different requirements depending upon what volcanic hazard affects them. There are also differing timescales as to when messages are issued and messages that are to specifically fulfil legal requirements.

During a crisis, each observatory follows a number of protocols, many of which are correlated to different alert levels. Once the scientist in charge makes the decision to change alert level or to issue new information (usually by consensus of the observatory staff), the first requirement is to conduct a call-down, that is, a sequenced set of quick telephone calls to key agencies to notify them in person of the information, and enabling discussion as to why the alert level has been issued and what the specific data and interpretation is. Call-down sequences are context specific, with each observatory using a sequence that depends on relevant local factors (including land owners, jurisdiction, the volcano’s activity and the specific hazard characteristics). Once the call-down is complete, an electronic form of the information is emailed to all addresses on relevant observatory mailing lists. Both call-downs and emails are multi-valent communication tools, providing opportunities for scientists and users to discuss the information behind alert levels. By contrast, uni-valent communication tools do not allow users to seek clarity and ask specific questions of scientists in real time.

These opportunities are important, since there are numerous hazards that can occur within close proximity of a volcano that are excluded from the VALS, which relates only to the occurrence of volcanic/eruptive unrest/activity. Some of these hazards occur irrespective of volcanic activity, in different locations and at different times. To address this issue, a number of the observatories have developed independent alert level systems for hazards that require warning systems specifically tailored to the nature of the hazard, such as volcanic gases (in particular seen at HVO), lahars (CVO), volcanic ash clouds, volcanic ashfall (AVO) and hydrothermal activity (YVO) (see further information in Fearnley (2011)).

## How VALS works in practice

Consistency is frequently identified as a key benefit of standardised hazard and risk communications. A narrow focus on consistency, however, fails to account for transactional and other factors contributing to the effective use of VALS in practice. VALS works well in operation, with all observatories using it to relay the status of volcanic activity. These practices rely heavily, however, on prior experience to familiarise all involved with the meaning of different VALS alert levels. Similarly, the limitations on the capacity of VALS to provide useful and usable information are mitigated by the largely multi-valent communication techniques and cross-boundary networks that have developed between scientists and VALS users. To appreciate and understand the workings of the standardised VALS model, in other words, requires that it be recognised as context dependent, relying heavily on everyday practice. That is, the new standardised VALS ‘works’ precisely because it continues to afford scientists the flexibility to conduct their usual communication practices in order to translate scientific information into a form that is accessible to bureaucratic rationalities.

Scientists and users commonly regard VALS as a catalyst that initiates the communication (both uni- and multi-valent) required to facilitate required discussions and operational decisions. As one scientist commented:

[An] alert level system is a shorthand, is the vehicle, it is the excuse to get into communications and dialogue, that gives you a justification and purpose […] that provides you the entry into having a discussion with very busy people who are otherwise occupied with other duties they have (VHP manager 4).

In practice, a VALS is a communication initiation tool, an instrument to develop coordination plans and to provide general awareness about the state of the volcano, rather than about a specific hazard. If this communication occurs regularly, then it may actually be surplus to requirements. That is, VALS can appear overly complicated given that the concept is simply to gain attention to an event and its anticipated impacts, and valuable time can be spent on deciding alert levels that might better be used to initiate the necessary communication to provide scientific information. It is through multi-valent communication outside of the VALS that producers and consumers can establish meaningful interpretations of warnings, even if they are based in different contexts.

**Fig. 5:**
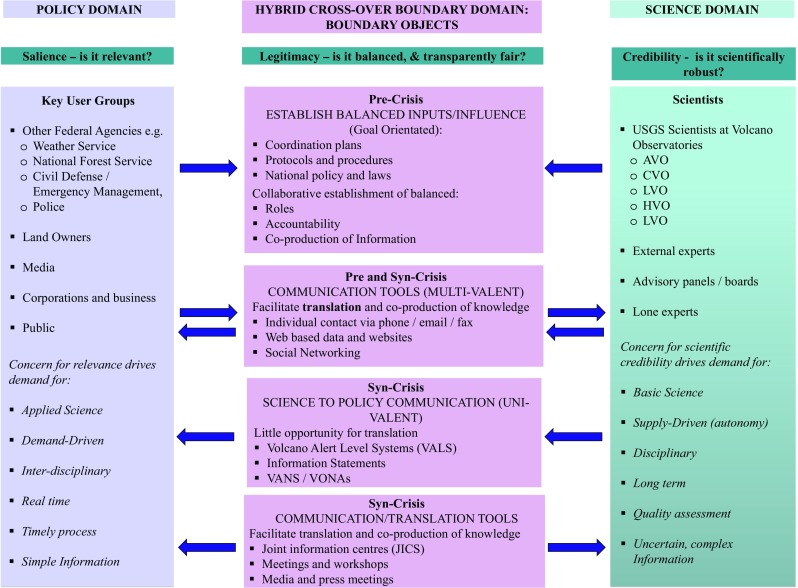
Mapping credibility, relevance and the generation of legitimacy to translate, communicate and mediate volcanic crisis information (adapted from Beaven et al. (2017), Parker and Crona (2012) and Sarkki et al. (2013))

Using Cash et al.’s (2003) model, we break down the various processes and issues that make the VALS and other communication tools work in practice by classifying them under (i) the need to ensure they are scientifically robust, driven by the scientific credibility requirement; (ii) the need to generate salient knowledge relevant to the needs of decision makers; and (iii) to need to ensure that (i) and (ii) are balanced, in order for both processes and information to be perceived as legitimate. This classification is summarised in Fig. 5.

### Credibility

To be successful, a VALS needs to meet scientific credibility criteria. However, there are a number of challenges facing scientists responsible for ensuring the accuracy of the warnings they provide, many associated with negotiating the uncertainty of the phenomena and of the monitoring data. In order to provide timely volcanic hazard warnings to communicate to the users, it is important for the scientists monitoring the volcano to accurately interpret scientific data, provide the best information about current activity and if required, generate reasonable forecasts for potential hazards; this is addressed fully in Fearnley (2013). Before scientists discuss what alert level volcanic activity should be assigned, there is a rigorous process of establishing exactly what is going on at the volcano. This process is often dependent upon the monitoring capabilities of each observatory to provide scientific data, the technology and staff to interpret this data and the need to form a consensus about what the volcano is doing. Whilst global databases such as WOVOdat and GVM bring together global expertise and comparative examples, each volcano has its own ‘personality’ (CVO senior scientist 7). Therefore, understanding a volcano is ‘part science, partly an art’ (CVO senior scientist 7), since volcanoes can behave in unexpected ways, and recognising patterns of behaviour for a particular volcano is critical to understand what that volcano is doing and to generate accurate forecasts. The difficulties of establishing a consensus concerning volcanic activity are further exacerbated by the often-short timeframes in which scientific evaluations need to be conducted during a crisis and by the fact that these evaluations often have a shared, rather than individual value. Although procedural protocols can contribute to the credibility of scientific evaluations, it is also important to recognise the subjective components in these evaluations.

Further complications arise when the scientists review possible forecasts. Forecasts are highly relevant to users. Forecasting volcanic behaviour involves much greater uncertainty than simply determining volcanic activity, since given volcanoes can sustain unrest or eruptions for long durations. These uncertainties make assigning a volcano alert level and deciding when to ramp-up or reduce the levels a difficult, highly complex and concerning process, with real-world consequences. Essentially, this ‘forces the scientist to think about the alert levels rather than the science’ (HVO senior scientist 5), driving a process in which scientists rank the possibilities of what is likely to happen and then release this information, rather than conducting in-depth scientific discussions. We concur with Papale’s (2017) concerns about the position this puts scientists in. We differ from him, however, in that we understand this position to be created by the use of a linear process to try to manage a highly complex situation, and decision-making processes.

### Relevance

For VALS to be effective, assessments conducted by scientists must be relevant to the needs of the key decision makers. The relevance requirement has been found to drive associated demands for timeliness and for simple accessible alert information (Sarkki et al. 2013; Parker and Crona 2012). With reference to VALS, this includes demand for timely simple and accessible alert information, that is usable subject to a range of contingent factors.

#### Timeliness

When providing warning information, the key issue for end-users is to provide *timely* information. During times of non-crisis, those involved can spend time deliberating plans and protocols. During a crisis, information is required quickly, regardless of scientific uncertainties, and with guidance on what the information means and how to act upon it. Scientists can be reluctant to disseminate guidance due to scientific uncertainty, since it is often impossible to accurately predict what a volcano is going to do until days/hours/minutes before it happens. In addition to concerns about the credibility of the information, scientists are also concerned about the legal context of such warnings. Numerous users reported in interviews that volcano observatories are discouraged from issuing alerts until there is greater certainty, because a VALS is a legal ‘formal warning’ under the USGS mandate provided by the USA Stafford Act. This places pressure on the scientists to get the decision ‘right’ before issuing alerts (AVO scientist 3).

Users however are much more concerned with the relevance of information. It was common for users to state that it is better to communicate what is known to the users irrespective of how certain or clear scientists were about the situation. For users, it is much better to say something albeit uncertain rather than ‘nothing at all’ (LVO scientist 2). A user in the Cascades, for example, expressed frustration with the time needed to debate and initiate a VALS and accompanying information statement,

Basically, after the action in 2004, I said I thought that it was dangerous actually; that they got state emergency managers and people like ‘x’ just sitting around deciding what the words [for the information statement] are going to say, and I said ‘you know we need to call ‘y’, and let them know. I don’t care what it [information statement] says we just need to know that something has changed’. To be sitting worrying about what the three sentences are is silly. […] There is this tension between wanting to have everything be just right and needing to get the word out (CVO user—USFS).

Another lesson here is that informal methods of communication such as telephone calls were a valuable means of facilitating timely interaction and the translation of scientific uncertainty one on one to end-users, since they did not technically involve issuing official warning information.

#### Establishing meaning in alert levels

The explicit aim of introducing standardised VALS, as noted above, has been not only to ‘fix’ the meaning of the information they convey but also to fix the meaning of a VALS itself. Such efforts are complicated by the fact that each VALS is understood in relation to prior learning experiences, that ‘school’ users to respond in particular embodied ways to the medium provided. One such example, recalled at both AVO and at HVO as illustrative of the role of prior experience, concerned a commercial Alaskan pilot flying from Alaska to Hawaii. The pilot, used to flying in Alaska and dealing with the aviation colour code frequently in place there, was concerned that the Kilauea volcano on the island of Hawaii was assigned an orange alert level. Based on his experience of warnings issued in Alaska, he anticipated that the volcano would be exhibiting unrest with increased potential for eruption with ash. When the pilot arrived in Hawaiian airspace, he expected some form of diversion or information (such as a Volcanic Ash Advisory) regarding Kilauea, but received nothing and landed with no problems. He later discovered that Kilauea was erupting, but emitting such a small ash plume that low-level flying was only prohibited within close proximity of the volcano.

Generating a meaningful warning or ensuring that information that is used as intended is challenging for a number of reasons. The ambiguity of alert levels is acknowledged. In particular, the orange/watch alert level presents a problem, because it involves two descriptions for the VALS, with one predicting imminent eruption and the other describing an already occurring but non-threatening eruption. This is a specific issue demonstrating the importance of VALS design and criteria. There is general concern that an alert level can generate complacency. If at a single status other than green/normal for too long, the alert level loses meaning and impact, as seen with the Homeland Security Terror Alert System which has been historically disregarded due to perceived lack of efficacy, often staying at one alert level for years rendering it meaningless (CVO user—emergency manager 2). For places like Hawaii, constant eruption means alert levels remain at orange/watch. For users, this can become the ‘status quo’, prompting them to think ‘I do not need to be worried about it’. In the case of pilots, this kind of thinking can put the lives of passengers in danger if the volcano suddenly erupts, as per the orange/watch alert level (AVO user—FAA).

### Legitimacy

Legitimacy relies on the perception that the processes through which information has been generated and disseminated have included and balanced the interests and knowledge of all involved (Cash et al. 2003; Sarkki et al. 2013). This can be difficult to do using VALS, since they are not a static tool but rather change in purpose as the volcano ramps up and down from an eruption, and during various phases of eruptive activity. VALS have in effect been used to encompass at least two roles, driven by demand from users, consisting of a forecasting tool with warnings and a reportage tool describing what was happening at the volcano, such as an eruption, also seen in Quito, Ecuador, in 1998 (Metzger et al. 1999). This combination of roles can help to make a range of users become more expert, increasing understanding of the history of the relevant volcano and awareness of what to watch out for, so that once an alert level is issued they know how to respond (AVO user—NWS 2).

The overriding consensus among all interviewees as to what VALS are intended to accomplish was that VALS were an awareness-raising medium, or ‘flag’, providing a “heads up, pay attention, something has changed, you need to look at reports, updates for information statements, or listen to advice from federal agencies” as one interviewee put it (CVO Senior Scientist 2). This applied across the interface between communities of experts and of end-users. The VALS helped to “ramp up situational awareness” among users in that it “dictates and drives our situation awareness and staffing” (AVO user—NWS 3), and provided an equivalent “flag for users for how often the users should be looking for information” (CVO scientist 12). Interviews suggested that scientists similarly responded to VALS as an awareness raising mechanism, since the rate at which they “process and disseminate information is dependent on the alert level” (CVO scientist 12), implying that at red alert, information statements would be issued faster than at yellow alert.

#### Expectations on the role of the VALS

Defining a VALS is critical to establish whether or not it serves its purpose and to determine whether stakeholders share a common understanding of what the alert levels are. The overriding consensus among the expert community was that VALS were used to alert users, communities, and individuals to the state of the volcano in a simple and concise message that allowed them to gear an appropriate response (AVO senior scientist 1/HVO scientist 1). Scientists regarded VALS as a tool to translate scientific assessment information about the nature of volcanic unrest and possible hazards quickly to non-scientists, excluding technical details, so that the use of one word, such as ‘Red’, would allow a large and diverse range of people to know what the conditions are (HVO scientist 2). At the same time, it was also widely accepted that an alert level alone cannot provide all the information required for users to make decisions:

[The] complicated reduction of all of these factors (risk, hazard, activity) and boiling that down to a simple number [means] inevitably if you do that, something is going to be lost. You can’t just project a ten-dimensional problem down to one dimension and expect it to retain all its complexity (AVO scientist 4).

How an alert level is defined and what it means depends on a balance between the meaning conveyed by the scientists and that understood by the users, which relies on their institutional requirements for the alert. Interviews with users suggested that they regarded the VALS as a scale to determine the importance and relevance of the information being distributed. This is at odds with the understanding of those issuing the VALS, who regarded it as a concise indication of the science—which is to say the eruptive activity of the volcano. Users tended to work in busy government agencies that were often already overwhelmed with other duties. This made establishing the urgency of warning information a key priority, since they needed to determine “where we are in terms of imminent danger” (LVO user—Mammoth Lakes town 2). The VALS helped to “ramp up situational awareness” and “dictates and drives our situation awareness and staffing” (AVO user—NWS 3). Consequently, although VALS provided information about the physical hazard, they were primarily useful and used for planning purposes. Some senior scientists felt that VALS were in effect a distraction from the need for more communication between scientists and non-scientists:

I think the whole alert level thing is […] an attempt to better communicate with the public, media [and] help scientists convey the message. Most people put too much emphasis on that and not enough with the basic problem, which is communication between scientists and non-scientists (HVO senior scientists 4).

User groups tended to agree with this assessment, but from the other direction. Those in charge of making decisions about people’s safety had difficult problems to deal with like “should I evacuate or not?” (LVO user—emergency manager 1), “where are people going to go/live?” and “will people actually pay attention or ignore the warnings?” (HVO user—emergency manager). Using the VALS alone cannot facilitate discussion of these real-world issues. Where scientists focused on the need to translate scientific information to users, emergency managers expressed the need for scientists to develop a better understanding of the problems they face in order to help with their decision-making processes, and limitations in knowledge (Fearnley 2013).

#### The role of contingency

Every volcano has a diverse range of hazards in different spatial and temporal combinations, making the individual behaviour of each unique. This can make understanding the activity and issuing a warning for a volcano alert a highly complex and context-specific process. Many hazards can occur within close proximity of a volcano, whether it is active or not, in different locations (geographically), and at different times. Most are excluded from the VALS, which relates only to the occurrence of volcanic/eruptive unrest/activity, and must apply to every volcano. Many scientists stated that VALS should convey information about *all* volcanic hazards, whether they proximal to the volcano, i.e. volcano-centric, or distal. Some expressed the view that a warning can only be truly issued after the event has begun (CVO collaborator 2), which means that the only way to measure if a lahar has developed, or where an ash cloud is moving, is to monitor them individually. A number of the observatories have developed independent alert level systems tailored to the nature of a range of these hazards, including volcanic gases (in particular seen at HVO), lahars (CVO), volcanic ash clouds, volcanic ashfall (AVO) and hydrothermal activity (YVO). The unique individual behaviour of a volcano, each with differing hazards in differing spatial and temporal relations makes monitoring, understanding the activity and issuing a warning for a volcano alert highly complex processes.

In addition, contingent institutional factors need consideration as they are shaped by local cultural, political and judicial systems. The dynamics of USGS policy, governance and operations had a profound effect on the resources required to provide an effective VALS, such as funding for monitoring capabilities, staff resources and protocols for issuing warnings. Education and outreach were essential activities to ensure that stakeholders are aware of VALS and how they work, but these required significant staff time and resources. In addition, each user group will also have their specific institutional and legal remits, factors and limitations to consider. Inevitably these factors, along with securitisation, influence how communities will respond to a warning.

The diverse range of contingencies arising out of the institutional and geophysical context associated with any given volcano further underlines the importance of including VALS as one among a suite of boundary objects used to facilitate the co-production of knowledge and decision-making about volcanic risk. To make sure that the decoding of a warning was accurate and meaningful, all the actors involved worked very hard external to the VALS. This was done using other boundary objects, including multi-valent communication products (via protocols) and collaboratively developed coordination plans and meetings that gave user groups opportunities to discuss what the hazards and potential risks were, and to prepare by co-producing response plans. This open, multi-valent communication allowed mutual understandings of events to emerge, helping to bring together knowledge and expertise from both sides of the science and decision-maker interface. It was this process that over time helped all those involved (including the observatory scientists) to recognise and understand each other’s needs and concerns, thereby incorporating local context into the dialogue process. Such a dialogue depended, however, on preparatory work in order to make the communication network ‘work’.

These communication techniques function differently from VALS. They fostered a sense of trust based on dialogue, for example, rather than implying the top-down authority created by the uni-valency of VALS. A number of users expressed the view that trying to get “facts out of scientists” was difficult, but by “building trust ahead of time”, it was possible to trust each other and understand each other’s limitations, despite institutionally “different cultures” (CVO scientist 5). This was also the conclusion of Peterson (1988, p. 1467) who pointed out after interviewing journalists and scientists following the 1980 Mt. St. Helen eruptions that journalists found that “scientists are too long-winded; they talk all around the subject and never get to the point; they do not understand that we need to use straightforward, simple statements; we have to convert the complicated discourses to words that people can read” (after Peterson 1988, p. 4167).

The development of coordination plans—as implemented in Alaska, the Cascades, Mammoth Lakes and in Hawaii (Hill et al. 2002, 2017; Madden et al. 2008)—was key to this process. The plans were drawn up to provide background information about the volcano, its history and potential hazards, the different land owners, stakeholders and federal or state agencies involved with the land, and the plan for a crisis. It is through meetings, developing preparedness plans and reconvening frequently that the actors get to know one another and create the potential to rapidly generate “situational awareness” between scientists and users (CVO scientist 5). This enabled scientists to communicate issues around uncertainty, for example, so that “they [users] have some sense of our level of anxiety on escalating unrest on a volcano so they can understand the alert levels better” (LVO senior scientist 1). Furthermore, coordination meetings provided the opportunity to clarify the nuances of the terms as understood by the users and scientists, so each group was confident about what each alert level is likely to mean to each user for that specific volcano.

## Conclusions and communications best practice

Analysing VALS as boundary objects that are successful to the extent that they satisfy the informational requirements of both scientific and end-user communities clarifies both the utility and limitations of VALS. To the extent that they are provided as information about a scientific event (volcanic unrest), but received as an indication of relevance (the comparative urgency of the information), VALS alerts do not translate science into a form that is understood by and relevant to end-users. Rather than constituting scientifically credible and relevant information, the alerts change from scientifically credible information to a different, relevant message, as they pass from scientist to decision-maker community. To be legitimate, the information conveyed by VALS needs to remain both scientifically credible and relevant on both sides of this interface. This requires that VALS be deployed as part of much wider discussion and consultation networks that bring scientists and users together early to facilitate effective, relevant hazard and risk decisions based on credible, widely understood science that is relevant to decision-maker needs. To be legitimate, VALS need to be perceived by all to include and balance the needs, interests and perspectives of scientists and decision-makers. This inclusion and balance was occurring through the discussion and consultation networks through which VALS were disseminated and enabled. But a stronger focus on developing the decision-making and communication processes that already occur outside of the current VALS so that they are more accessible and transparent would enhance the legitimacy of information further, by providing transparent access to the processes of information production. This would also make it possible to simplify VALS, reducing its limitations and potentially improving effectiveness as one among a number of boundary-spanning tools.

VALS are used globally and provide a general level of awareness that volcanic unrest is occurring, despite inabilities to be specific. The research presented here suggests that simplifying a VALS to minimise limitations would be beneficial for all stakeholders. GNS in New Zealand, for example, recently redesigned their entire alert level system into six levels based only on volcanic phenomenon and likely associated hazards (Potter et al. 2017). Using only volcanic phenomenon that ranges from small-scale unrest to caldera-sized eruptions, this system has been designed to make the assignation of the alert level easier and quicker by clearly defining simple descriptive levels with no elements of forecasting, risk or multiple meanings. Such a considered VALS has the potential to minimise complications relating to the issuance and interpretation of alerts, and it will be of interest to see this new VALS operates following a number of crisis.

**Fig. 6:**
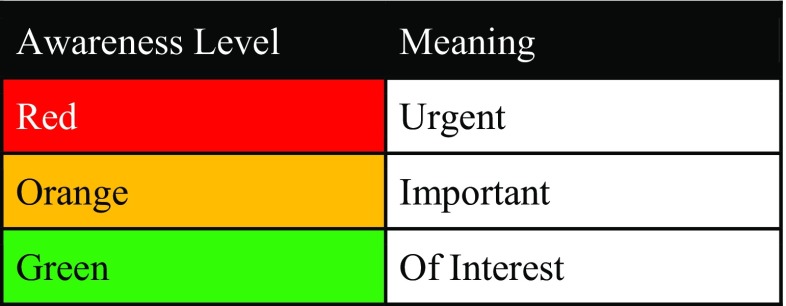
An example of what a Volcano Awareness System could look like (Fearnley 2011, p. 256)

But are there alternatives? Given that the emphasis on scientific certainty is hampering the capacity to issue timely warnings, it may be time to adapt VALS to reflect the informational requirement of users for timely relevant information. A system that was devised to show how severe/urgent a warning is and how much attention is needed by user groups would be much more relevant to their informational requirements, and easier to issue in a timely fashion. This is critical for uni-valent communication tools such as information statements. Again, however, in order to ensure that it was both relevant and scientifically credible, communication tools would have to be developed through transparent communication and discussion networks that bring scientists and end-users together so that all involved understand the processes through which the alert information is developed. Or as Peterson (1988) put it “we must draw deeply into our reserves of creativity and ingenuity to find more effective ways to present essential information, perhaps by improving our own presentation, perhaps by finding new allies to assist us” (p. 4168). Such a VAS (see Fig. 6) could serve a similar purpose to a VALS, but in a more transparent way—based on science, and acting as a trigger for discussion via the already existing communication structures so that users can make decisions based on timely information (even if uncertain), and the scientists can focus on providing this information and interpreting the scientific data. Colours are deployed here as opposed to weather related terminology (see more about alerts used in weather forecasts in Gill 2008), because users have been schooled to regard these as indicative of various levels of urgency.

Whilst large volcanic eruptions gain the attention of the media, many people who live around active volcanoes in the USA are affected by hazards that persist over long periods, such as noxious gases (e.g. Long Valley caldera), and low-level seismicity (e.g. Long Valley, Yellowstone and Hawaii). Such ever-present hazards are not captured in the VALS, despite providing discomfort to local populations. A Volcano Awareness System could help accommodate short to medium-term changes and indicate the level of hazard/risk at each volcano and the anticipated severity of a hazard such as a lahar, ash or gas emissions to user groups. Removing both the alert level descriptions and the focus on the eruptive activity enables the system to express awareness about the different hazards and situations in a simple design. This system could potentially be standardised nationally, potentially internationally, yet be locally operated and adapted for the local hazards and needs, and reflect temporal changes effectively.

It is important to again highlight that current knowledge and research on VALS remains limited. It is not yet possible to outline appropriate or inappropriate use of VALS or to establish guidelines, in part because each VALS and institution using them is driven by their unique, and changing set of contingencies. The VALS Working Group as part of WOVO are working to gather vital information and empirical data on VALS that is much needed to answer many outstanding issues about the use and effectiveness of VALS.

The research and work presented in this paper makes it clear that the capacity to identify issues and solutions during volcanic crises relies on dialogue and collaboration between scientists and those at risk. This is not to suggest that scientists be required to make decisions on non-scientific issues; as Papale (2017) argues, this would be inappropriate. Rather, it is to argue that such decisions should be the result of deliberative processes. It is clear that whilst VALS are still evolving, they do have a pivotal role in creating the common ground to bridge the gap between the scientific and the operational side of volcanic crisis management. Bridging this gap is necessary to foster the interaction required to manage volcanic crises. In terms of best practice, it is recommended that rather than attempting to ‘police’ the divide between hazard (observatory scientists) and risk (user groups), sustained dialogue and trust building is key to ensuring that outcomes are both scientifically credible and relevant, and so generating the legitimacy required by all stakeholders involved in the management of volcanic crises.

## Footnotes

1 Information on the VALS Working Group can be found here: https://vhub.org/groups/volcanicalertlevelworkinggroup

2 FOIA stands for The Freedom of Information Act in the USA where any person has the right to request access to federal agency records or information except to the extent the records are protected from disclosure by any of nine exemptions contained in the law or by one of three special law enforcement record exclusions.

3 In the following account, data has been anonymised, but the observatory and details of the job specifics are stated in quotes to provide necessary context.
